# Innovative flavoring behavior in Goffin’s cockatoos

**DOI:** 10.1016/j.cub.2025.01.002

**Published:** 2025-02-10

**Authors:** Jeroen Stephan Zewald, Alice Marie Isabel Auersperg

**Affiliations:** 1 Comparative Cognition, Messerli Research Institute, University of Veterinary Medicine Vienna, Medical University of Vienna, and University of Vienna, Veterinätirplatz 1, 1210 Vienna, Austria

## Abstract

Dunking behavior can be a foraging innovation in non-human animals in which food is dipped in a medium prior to consumption.^1^ Five functions of this behavior have previously been suggested (soaking, cleaning, flavoring, drowning, and transporting liquid).^2–8^ Although experimental reports exist,^1,5,9–11^ most dunking observations are anecdotal,^12^ making it hard to infer its function. Previously, we reported innovative dunking behavior in a group of Goffin’s cockatoos (*Cacatua goffiniana*) with the apparent function of soaking dry food.^13^ Here, we report cockatoos dunking in soy yogurt with the likely function of flavoring their food, something thus far only observationally reported in Japanese macaques.^3,14^ In an experimental setup with two types of soy yogurt and water, 9 out of 18 cockatoos dragged food through yogurt, with an overall preference for blueberry-flavored yogurt over neutral yogurt, which could not be explained by color preference alone. Furthermore, the cockatoos showed an overall preference for the combination of yogurt and noodles in a separate food preference task. This combination of quantitative and qualitative results indicates that the cockatoos use yogurt to flavor their food, preferring this combination rather than the yogurt flavor alone. Considering that not all cockatoos dunk their food in yogurt, and little overlap in individuals dunking in a previous study,^13^ this suggests a second food preparation innovation in this species. Our results thus provide experimental evidence of innovative food flavoring behavior outside the primate lineage, which may supplement our present understanding of the emergence of rare forms of food preparation behaviors in animals.

## Results and Discussion

### Yogurt dunking behavior and its function

**Figure 1:**
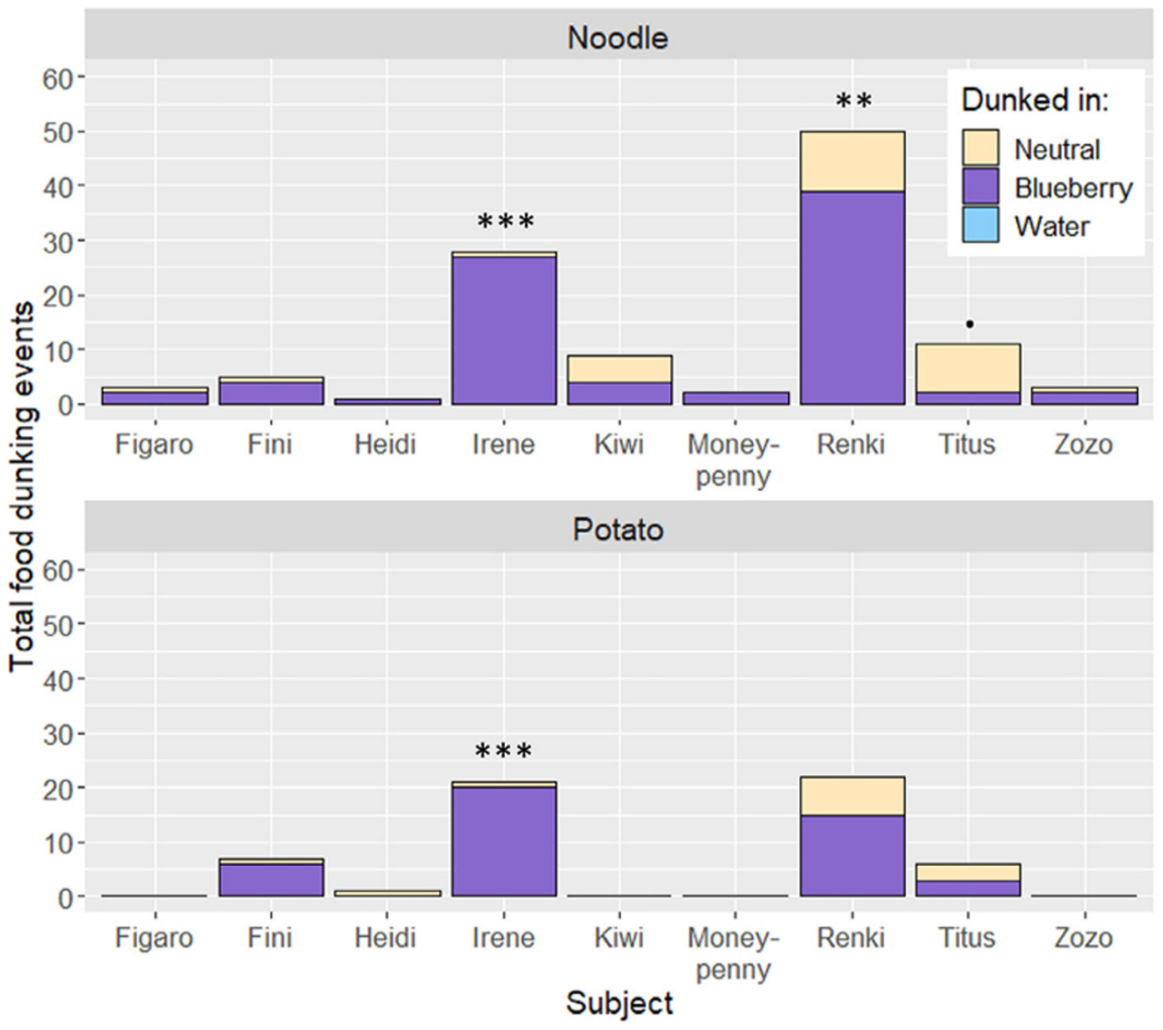
The total dunking events over all breakfast sessions per subject The upper panel shows the dunking events with noodles as food and the lower for potatoes. The color represents the medium that the food was dunked in. The significances of the individual preferences (binomial test) are represented with: *p* < 0.1, * *p* < 0.05, ** *p* < 0.01, *** *p* < 0.001. See also Figures S1–S4, Table S1, and Videos S1, S2, and S5.

In November 2022, two cockatoos (*Irene* and *Renki*) were incidentally seen dunking cooked potato pieces into blueberry-flavored soy yogurt during breakfast at the Goffin lab in Austria. Continuing our previous dunking observations,^13^ we investigated the function and frequency of this behavior. Therefore, we conducted 14 additional breakfast observations (30 min each), in which we presented the group of cockatoos with a food bowl and three potential dunking mediums: (1) fresh water, (2) blueberry-flavored soy yogurt, and (3) neutral soy yogurt, which acted as an unflavored texture control (see Data S1 for yogurt details). 9 out of 18 cockatoos dunked food into yogurt (Video S1; Table S1). Carrots and cauliflower were never dunked (and rarely eaten, Figure S1). On average, noodles (mean per individual ± SD: 12.44 ± 16.36 events) were dunked more often than potatoes (6.33 ± 9.01 events) (Poisson generalized linear mixed model [GLMM]: X^2^_(1)_ = 18.23, *p* < 0.001). The cockatoos never dunked food into water. However, they did show a higher probability to dunk food in blueberry yogurt than neutral yogurt (binomial GLMM: 0.73 ± 0.62, z = 2.05, *p* = 0.040; Figure 1). This probability did not significantly differ between potatoes (0.73 ± 0.10) and noodles (0.71 ± 0.12) (binomial GLMM: X^2^_(1)_ = 0.04, *p* = 0.843). The cockatoos also had a higher probability to eat from the blueberry than the neutral yogurt (binomial GLMM: 0.93 ± 0.70, z = 3.01, *p* = 0.003, Figure S2). Thus, the cockatoos seemingly dunked their food more often in their preferred medium.

**Figure 2:**
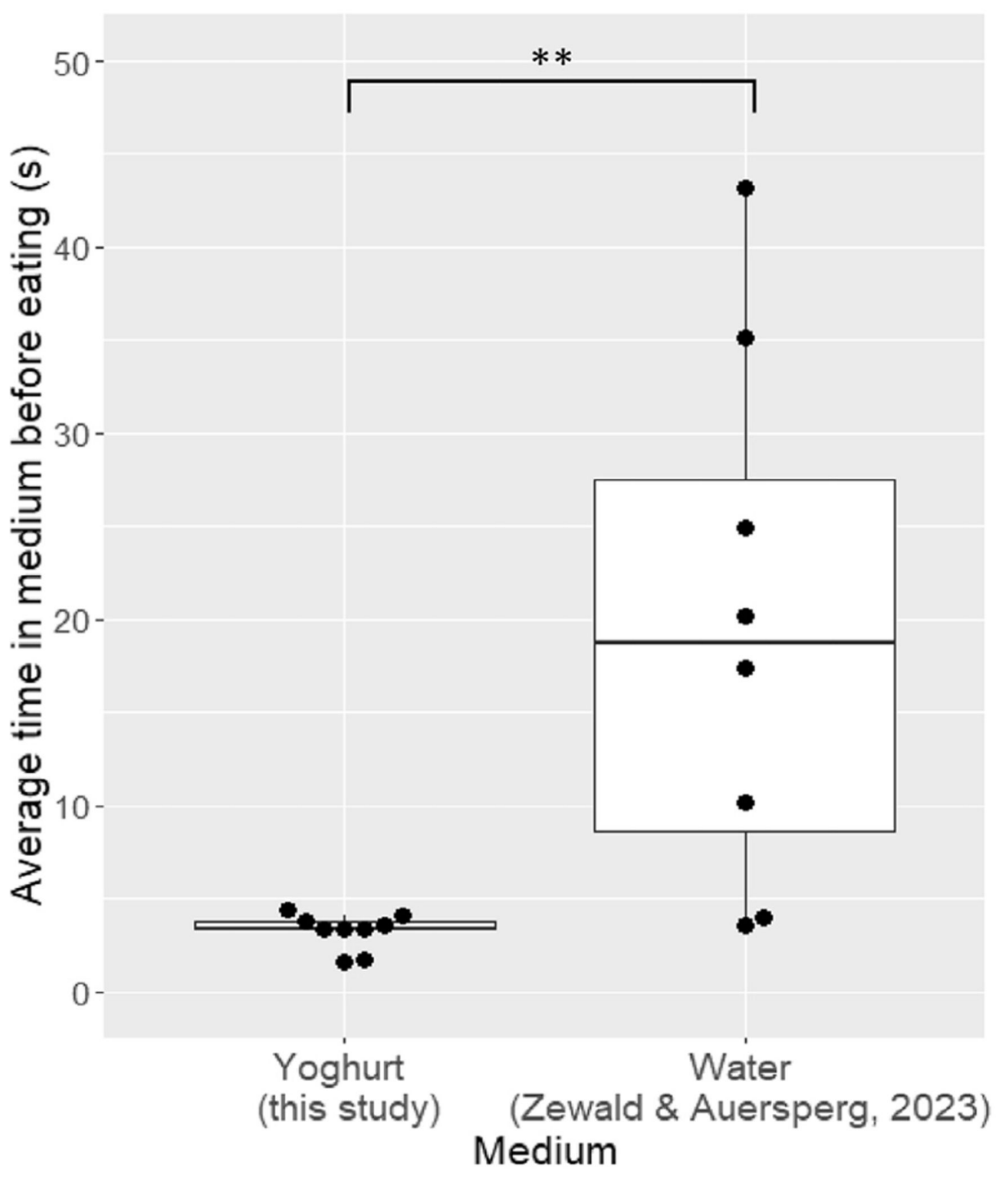
The median time the food was left in the medium for yogurt dunking versus rusk soaking These boxplots show the dunking times (s) for this study in yogurt and for the previous study in water.^13^ Each dot represents an individual, with some horizontal variation for the visualization. The boxplots present the median and interquartile ranges. *p* value is represented with * *p* < 0.05, ** *p* < 0.01, *** *p* < 0.001.

In the literature, five functions of dunking behavior have been suggested: soaking,^1,2,13^ cleaning,^5,9–11,15^ flavoring,^3,14^ drowning prey,^6,7^ and liquid transport via food-containing liquids.^4,8^ First, we can rule out drowning prey, as there is no living prey involved. Second, cleaning the food seems counterintuitive as the food was clean, the birds never dunked it in water, and they added a substance instead of removing one. Third, the birds may have combined the foods to take them elsewhere to eat without social interference (reminiscent of the transport function^4^). However, they did not show a higher probability to eat dunked food near the bowl (<20 cm away) or elsewhere (binomial GLMM: 0.33 ± 0.68, z = −0.934, *p* = 0.35, Figure S3), making the liquid transport function unlikely. Similarly, the food could be used as a tool to facilitate eating yogurt.^16,17^ However, this also seems unlikely as the birds still ate yogurt separately (Figure S2) and never licked off the yogurt before eating the combination (Video S2). Furthermore, if yogurt-eating facilitation was the goal, we would expect similar levels of dunking between the different food items, but, instead, the cockatoos showed a preference to dunk noodles more than other food items. Lastly, the cockatoos could dunk to soak their food.^1,2,13^ On average, the cockatoos left their food in the yogurt for 3.2 s ± 0.9 (Table S1), significantly shorter than the birds left the rusk to soak in water, as measured in a previous study^13^ (average of 22.9 s ± 25.5, Figure 2, LMM: t = 5.99, *p* = 0.008). With the food already being boiled and soft, and never being dunked in water, the function of soaking is unlikely as well.

**Figure 3:**
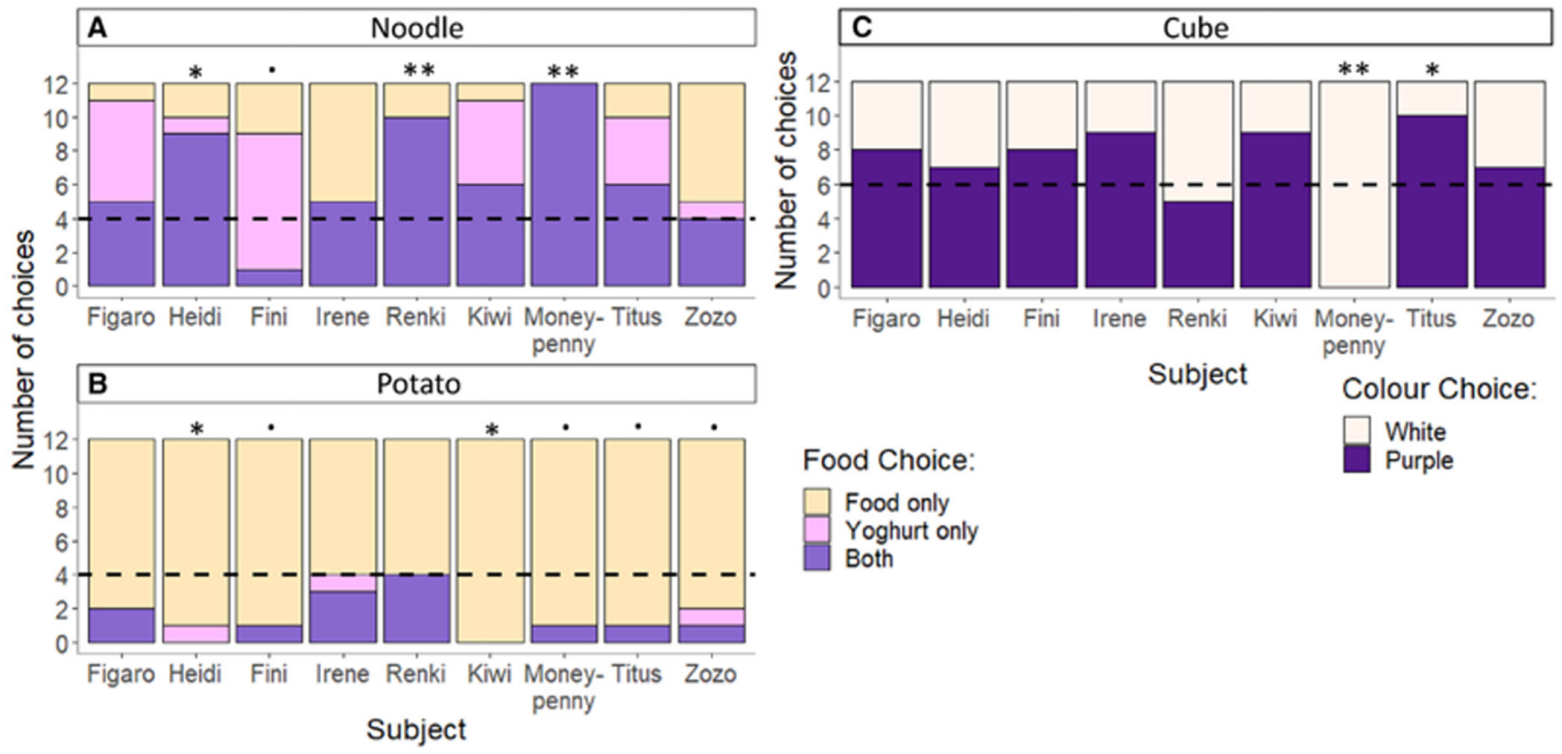
Food and color preferences for each individual The food choices made during the food preference test are shown for the noodles (A) and the potato pieces (B). The significance shows whether the individual had a significant preference or dislike for the combination (“both”). The color choices made during the color preferences are shown in (C). The color represents the choice made. The dotted lines represent the chance levels (1/3 for food choice, 1/2 for color choice). The significances of the individual preferences (binomial tests) are represented with: *p* < 0.1, * *p* < 0.05, ** *p* < 0.01, *** *p* < 0.001. See also Videos S3 and S4.

A more likely alternative function is flavoring, supported by their preference to dunk food in the blueberry yogurt over the neutral yogurt (Figure 1). To exclude that this preference was based on color only, we conducted a separate color preference test in which we presented the cockatoos with two cubes of similar colors to those of the yogurts (Video S3). Overall, the birds’ probability of choosing one color over the other was not significantly different from chance (binomial GLMM: 0.59 ± 0.59, z = 0.935, *p* = 0.35; Figure 3C), although we acknowledge that this color preference test is out of the foraging context. This was a deliberate choice because we want to continue to observe the spread of this behavior through our group in the future and altering the color association with these familiar food items directly would disrupt this. Nevertheless, our current color preference test helps to support the notion that their dunking foraging preference could not be explained by mere color preference alone.

More likely, the cockatoos preferred to dunk in this yogurt due to certain flavor attributes of the food combination. The blueberry yogurt had more sugar and more blueberry in it (see Data S1 for yogurt details), which the birds might have preferred to flavor their solid foods. Being able to perceive this extra nutritional value may give cockatoos an evolutionary fitness benefit. Although birds in general are said to have fewer taste buds than mammals^18–20^ and seem to have lost some sugar receptors in their early ancestry,^21^ recent studies have found other taste receptors to have evolved to perceive sugar in nectar- and fruit-feeding bird clades.^22,23^ Although this remains unclear in the Psit-taciformes clade, some species of Cacatuidae seem sensitive to sugars,^18,24^ so it is possible that these cockatoos also have this perception ability.

### Combination preferences

Qualitatively, the yogurt-dunking behavior looked different than previous dunking observations in the same group. When soaking rusks in water, the cockatoos would drop the food and wait (22.9 s ± 25.5) for it to absorb water^13^ before eating it. When dunking food in the yogurt, most individuals pressed, rolled, and dragged the food through the yogurt without letting go, which resulted in more yogurt on the food (Video S1). After that, they usually started eating the yogurt-covered parts of the food (Video S2), sometimes even re-dunking it after most yogurt was gone. They ate the food and yogurt together and never licked the yogurt off before eating the food, indicating their preference for the combination of both food items.

To see whether the dunking birds preferred this food combination over the food only or blueberry yogurt only, we presented them with a three-choice task between these items (Video S4). We found a significant difference in their probability of choosing the combination between the potatoes and noodles (binomial GLMM: 0.91 ± 0.60, z = 0.52, *p* < 0.001). For the noodles, the overall probability of choosing the combination was slightly but significantly above chance (binomial GLMM: 0.54 ± 0.08, z = 0.52, *p* < 0.001), although this was significantly below chance for the potatoes (binomial GLMM: 0.10 ± 0.04, z = 0.41, *p* < 0.001; Figures 3A and 3B). This preference may explain why the cockatoos dunked potatoes significantly less than noodles. That could be because (1) the combination of potatoes with yogurt is less tasty than with noodles or (2) the potatoes hold the yogurt less well than the spiraling structure of the noodles (fussilini). However, this does reflect the specificity of the dunking behavior to only a few food items and not just any combination, which would typically be expected if the goal was to only increase the nutritional value of all food items with the sweet yogurt. It thus seems that the cockatoos prefer the specific combination of the solid food and the blueberry yogurt rather than just the flavor of the yogurt alone.

### Foraging innovation

Previously, Zewald and Auersperg^13^ argued that rusk soaking in water by the same group was likely innovative because it was limited to a few individuals instead of being expressed by all cockatoos. Once again, we found that only 9 out of 18 cockatoos dunked food in yogurt (Table S1). Furthermore, dunking behavior has not been observed in these cockatoos in the wild (potentially due to a scarcity of opportunities to encounter open water/medium sources; B. Mioduszewska and T. Rössler, personal communication). Therefore, this does seem to indicate an innovation, although we cannot exclude that the lack of dunking in other individuals is due to individual preferences. The dunking cockatoos consisted of 5 males and 4 females across a range of ages (3–7 years old), making sex or age effects unlikely (although we could not statistically support this due to our small sample). Interestingly, however, only two individuals (*Kiwi* and *Moneypenny*) dunked both rusk in water in the previous study^13^ and food in yogurt in this study, whereas all others only dunked in one medium (either water or yogurt). These two individuals also seemed to have a different dunking technique than the only-yogurt-dunking individuals (Video S5). Although other individuals dragged and pressed their food in the yogurt, these two individuals dropped the food in the yogurt, picked it up, and repeated this to get yogurt on all sides, resembling the soaking behavior.^13^ This could reflect two separate dunking innovations or reflect a path to how a second function could have been discovered. Alternatively, because the food and the soy yogurt are presented in the same bowl during normal feeding, they may have accidently got mixed while eating, which might also have led to this discovery. However, we do not have data on this behavior before the start of this study, thus we cannot speculate who initially started this dunking and whether it was innovated by multiple individuals or socially transmitted.^25^ However, we will investigate the spread of this dunking behavior in this group to see whether this innovation will be picked up by other individuals as well.

Nonetheless, innovations of new foraging techniques like this have been correlated to various cognitive traits and shown to be a consistent predictor for residual brain size.^26–30^ Correspondingly, pallium neural number in Goffin’s cockatoos has confirmed that this species is highly encephalized, at a comparable level to other species renowned for their cognitive performance,^31^ like African gray parrots (*Psittacus Erithacus*).^32^ Furthermore, this active action of taking the food to the yogurt to dunk reflects other cognitive abilities, like delay of gratification, sequential problem solving, and a rudimentary form of planning, which have been previously found in Goffin’s cockatoos.^33–35^ This innovativeness may benefit the Goffin’s opportunistic lifestyle as a generalist (feeding on fruits, roots, and seeds) and as an island species with changing environments,^36^ complementing the cognitive buffer hypothesis.^37^ Future studies could investigate whether dunking behavior could develop in the wild, especially in the more recently introduced population in the urban areas in Singapore,^38^ where these artificial human-made food items might be more habitually consumed by the birds.

In conclusion, we provide evidence for innovative food flavoring behavior in a group of captive cockatoos. To our knowledge, this flavoring behavior has only been reported once, i.e., in Japanese macaques who dunked their food in salt water.^3,14^ Unfortunately, this early report was not further investigated with controlled experiments nor quantitatively analyzed, so alternative explanations for the saltwater preference (e.g. freshwater brook drying up or the provision of the sweet potatoes closer to the sea shore^3,14^) could not be excluded. Our captive setting, however, allowed us to experimentally control for many alternative explanations, with our results pointing to the function of food flavoring. Moreover, we were able to show two different types of dunking innovations (soaking and seasoning) in the same group of cockatoos. Following observational reports in Japanese macaques, we thus provide the first experimental evidence for food flavoring in animals. Our results will thereby help to supplement the sparse existing literature on food preparation behaviors in non-humans.

## Resource Availability

### Lead contact

Further information and requests should be sent to the lead contact, Jeroen Zewald (jeroen.zewald@vetmeduni.ac.at).

### Materials availability

This study did not generate new unique reagents.

See also Videos S3 and S4.

## Star*Methods

Detailed methods are provided in the online version of this paper and include the following:

• KEY RESOURCES TABLE
• EXPERIMENTAL MODEL AND STUDY PARTICIPANT DETAILS
  - ○ Subjects & housing
  - ○ Ethics statement
• METHOD DETAILS
  - ○ Dunking observations
  - ○ Food preference test
  - ○ Colour preference test
• QUANTIFICATION AND STATISTICAL ANALYSIS

## Star*Methods

### Key Resources Table

**Table:** 

REAGENT or RESOURCE	SOURCE	IDENTIFIER
Experimental models: Organisms/strains		
Goffin’s cockatoos (Cacatua goffiniana)	Goffin lab, University of Veterinary Medicine Vienna	https://www.vetmeduni.ac.at/en/cognition/goffin-lab
Software and algorithms		
BORIS v. 7.12.2	Friard and Gamba^39^	https://www.boris.unito.it/
R v. 4.3.1	R Development Core Team^40^	https://www.R-project.org/
Other		
Smartphone - Samsung Galaxy A52	Samsung	https://www.samsung.com/at/smartphones/galaxy-a/galaxy-a52-5g/
Alpro© Soy yoghurt - Natural without sugar	Alpro	https://www.alpro.com/at/produkte/soja-joghurtalternativen/soja-joghurtalternativen/natur-ohne-zucker/
Alpro© Soy yoghurt - Blueberry	Alpro	https://www.alpro.com/at/produkte/soja-joghurtalternativen/soja-joghurtalternativen-geschmacksvariation/heidelbeere/
R script, R workspace and dataset	Authors	https://doi.org/10.17605/OSF.IO/QAK6T

### Experimental Model and Study Participant Details

#### Subjects & housing

Our observations were done at the Goffin lab in Lower Austria, where a group of 18 Goffin’s cockatoos (*Cacatua goffiniana*, 9♀, 9♂, between the ages 3 to 13 years old, for details see Table S1) were housed in an enriched aviary (indoor: 45 m2, 3–6 m high; outdoor: ca 200 m2; 3–4.5 m high). During winter, the inside aviary is warmed to 20 °C and a 12:12 h light dark cycles is in place. Breakfast was served around 11:00 consisting of a weekly cycle switching between scrambled eggs, cooked potatoes and carrots with a teaspoon of palm oil, cooked cauliflower and noodles (fusilli, whole grain), and parrot cook mix (Birds and More Hungenberg Kochfutter) mixed with HIPP© baby fruit mesh. The food was always served alongside a few spoons of soy yoghurt (of various flavours) and fresh fruit. Lunch was provided around 14:00 consisting of bird pellets (Versele-Laga Nutribird© P15 Original), dried berries, dried banana and coconut chips, rusk, seeds, and supplementary minerals.^13^ Both breakfast and lunch were provided in ceramic bowls (Ø30 cm, 5 cm). Water for drinking and bathing was always available ad libitum and was provided in plastic tubs (Ø50 cm, 20cm).

#### Ethics statement

Our observations did not interfere with the normal feeding routine of the birds and were therefore considered as non-invasive and are thus classified as non-animal experiments following the Austrian Animal experiments Act (§2. Federal Law Gazette no. 501/1989). Furthermore, all animals included in this study were housed according to the Austrian Federal Act on the Protection of Animals (Animal Protection Act—TschG. BGB1. I no. 118/2004).

### Method Details

#### Dunking observations

We recorded 14 breakfast servings (7x potatoes and carrots; 7x cauliflower and noodles) from December 2022 to January 2023. During the servings, we placed three ceramic bowls in a row with 0.5m in between them (Figure S4). The central bowl contained the main food and the other two the yoghurts. 0.5m behind the central bowl, we placed the water bowl. We used two types of yoghurt: 1) neutral yoghurt (Alpro© Soy yoghurt - Natural without sugar), and 2) blueberry yoghurt (Alpro© Soy yoghurt - Blueberry) (see Data S1 for yoghurt details). Both yoghurts were regularly given to them during breakfast. The neutral yoghurt functioned as a control for the texture of the yoghurt without the flavour. The locations of the yoghurt types were pseudo-randomised between the two bowls. We video recorded the first 30 minutes of breakfast with a smartphone (Samsung Galaxy A52) and analysed the entire recording using the observational software BORIS.^39^ We analysed which individuals were dunking, how often, which food items they dunked, in which medium and how long they left the food in the medium. We also noted when the individuals ate the food or the yoghurt separately and if they ate it near the bowls or somewhere else (for ethogram see Table S2).

#### Food preference test

We individually tested the 9 dunking birds in a three-choice task (food only, blueberry yoghurt only, combination of both) before breakfast was served to investigate their food preferences separately. This was done in our testing room (7.5 m2, 3 m high) adjacent to the main aviary, where we could temporarily, visually separate an individual by calling it in, a common procedure to the birds. Therefore, participation was voluntary and if the birds showed any signs of distress (which did not occur), we would immediately release them back into the group. First, to make sure the birds were familiar with the three food options and the setup, they got 6 trials (2 trials per food item) in which we presented only one of the three food items (food only, yoghurt only or food covered with yoghurt) on a table (75×75 cm) in which they had to start eating the food item within 30 seconds to succeed, otherwise the trials were repeated on another day. After this familiarisation phase, the three-choice task started (Video S4). The experimenter placed the three food items on the table with an equal distance (70cm) to the chair, whose back acted as the starting perch. The food positions and order of placement were randomised, and the experimenter wore mirrored glasses to prevent cueing. The bird was then put on the chair and was given a ‘wait’ command for 2s after which it was given a ‘start’ command. It could then take one of the food items to eat, after which the other items would be removed immediately. If no choice was made within 1 minute, the trial would be terminated and repeated (which only happened twice). In total, each bird was given 24 trials (12 with potatoes, 12 with noodles) spread out over 4 days.

#### Colour preference test

We individually tested the 9 dunking birds in a two-choice task to test for colour preferences. For this, we used two wooden cubes (4cm x 4cm) in a colour closely approximating that of the yoghurts (Figure S5). We used the same setup as in the food preference test except for an additional flat wooden board on the table, to ensure the contrast between the cubes and the surface was equal for both colours. During the familiarisation phase, the birds were habituated to the wooden board after which one cube was placed in the middle of the board (the starting colour was randomised). The birds were asked to give the cube to the experimenter using the known ‘give’ command (an open hand gesture) within 1 minute, after which it would receive half a cashew nut and verbal praise. For the next trials, the colour of the cube would be alternated. In total, each bird would need to pass ten of these trial in a row (5 for each colour spread out over two days) to start the test phase. During the test phase, the birds were presented with both coloured cubes (placement location and order randomised) with equal distances to the chair (75cm; Video S3). After the first touch, the experimenter gave the open hand command in the middle of the two cubes, and the bird could place the object in the hand after it would be rewarded regardless of the colour choice. The birds got 12 trials in total over two testing days. After the 6^th^ trial, we gave two familiarisation trials (one per colour) to remind the birds that both cubes would still be rewarded.

### Quantification and Statistical Analysis

Our statistical analyses were done using R^40^ (version 4.3.1). For all Generalised Linear Mixed Models, we used the glmer and lmer functions of the lme4 package^41^ (version 1.1.34). First, to analyse if the birds dunked some food types more than others, we ran a Generalized Linear Mixed Model (GLMM)^42^ with a poisson error structure and a log link function. As predictors, we included the factor Food type (levels: Potato, Noodle) as a fixed effect and Subject as random intercept effect as we had repeated measures. Using a function provided by Roger Mundry, (2023; available on request), we assessed the overdispersion parameter for this model was to be close to 1 (0.948).

To test the other preferences of the cockatoos we ran 5 GLMMs with a binomial error structure and a logit link function. To investigate these preferences/proportions in these models, we used two-columns matrixes with the number of ‘successes’ and ‘failures’ for each subject as the response. For the responses of the first three models were 1) the proportion of eating events of the blueberry yoghurt and of the neutral yoghurt, 2) the proportion of eaten dunked food items at the bowl (<20m away) and eaten elsewhere (>20m away), and 3) the proportion of purple and white choices in the colour preference test. In these three models we only had the intercept as a fixed effect as a test for significant deviation from chance level (0.5) and Subject as a random intercept effect for repeated measures. For the last two models we used 4) the proportion of dunking in blueberry yoghurt and dunking in neutral yoghurt and 5) the proportion of choices for the combination of the yoghurt and the food and the choices for the food only, as response variables. Both these latter models, we included Foodtype (levels: Noodles, Potatoes) as a fixed factor and Subject as a random intercept effect. For both models, the full-null model comparison was significant (respectively, χ^2^_(1)_ = 48.766, p<0.001; χ^2^_(1)_ = 48.766, p<0.001). For the fourth model, we used the emmeans package^43^ (version 1.8.9) to get the probabilities of dunking in blueberry yoghurt over neutral yoghurt for each food type separately. For the fifth model, we readjusted the p-value of the intercept to see if it significantly differed from the chance level of 0.3333, as there were three potential choices (food only, yoghurt only or the combination). To see if each individual had a significant preference as a dunking medium, for all five binomial models, we used binomial tests and a Holm-Bonferroni p-value adjustment^44^ for multiple testing. For all these tests we used a chance level of 0.5, except for the food choice experiment in which we used a chance level of 1/3.

To compare the time the cockatoos left their food in a medium between this study and the previous dunking study,^13^ we used a LMM with a Gaussian error distribution. As response we used log-transformed time the birds left the food in a medium before eating it, the factor study (levels: this study^13^) as a predictor and subject as a random intercept as two birds were present in both datasets. We visually inspected QQ-plots^45^ to see if the assumptions for this model were met, which they were.

For all our models, we assessed the model stability by dropping each individual from the data one at a time and comparing the estimates derived for models fitted to these subsets with those obtained for the full data set (using a function provided by Roger Mundry, 2023; available on request) and the ‘Best Linear Unbiased Predictors’ (BLUPS) for the random intercepts.^42^ All models showed a robust result and the BLUPS were approximately normally distributed relative to our low sample size.

All plots were made using the ggplot2 package^46^ (version 3.5.0).

## Supplementary Material

Supplementary Material

## Data Availability

Our dataset, R code, and R workspace are publicly available and can found at https://doi.org/10.17605/OSF.IO/QAK6T. Any additional information required to reanalyze the data reported in this paper is available from the lead contact upon request.

## Highlights

- Foraging innovations, like dunking food, are linked to various cognitive measures
- We observed cockatoos dunking food in soy yogurt to add flavor to the food
- We used experimentally controlled tests to rule out alternative explanations
- This is the second, functionally different, dunking innovation in this group
