# Supplementary material for: Innovative flavoring behavior in Goffin’s cockatoos
Source: Curr Biol. Author manuscript; Available in PMC 2025 May 10. (PMC7617653; doi:10.1016/j.cub.2025.01.002)
Supplement: Supplementary Material [file EMS204743-supplement-Supplementary_Material.zip › 1-s2.0-S0960982225000028-mmc1.pdf]

**Current Biology, Volume *35***

## Supplemental Information

# Innovative flavoring behavior in Goffin's cockatoos

**Jeroen Stephan Zewald and Alice Marie Isabel Auersperg**

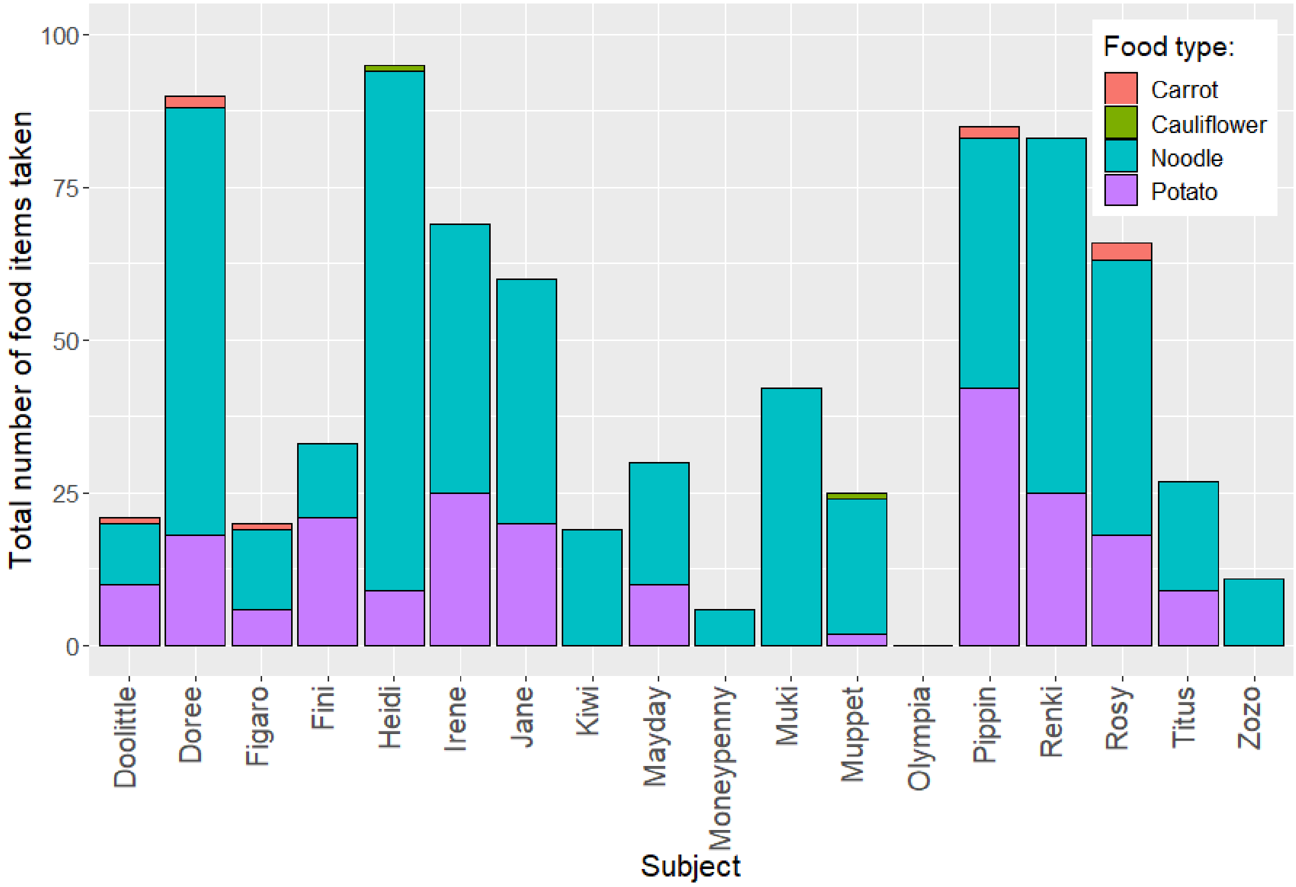


**Figure S1. Number of food items taken for each individual. Related to Figure 1.**
The total number of food items taken during all breakfast sessions. The colour represents the type of food.

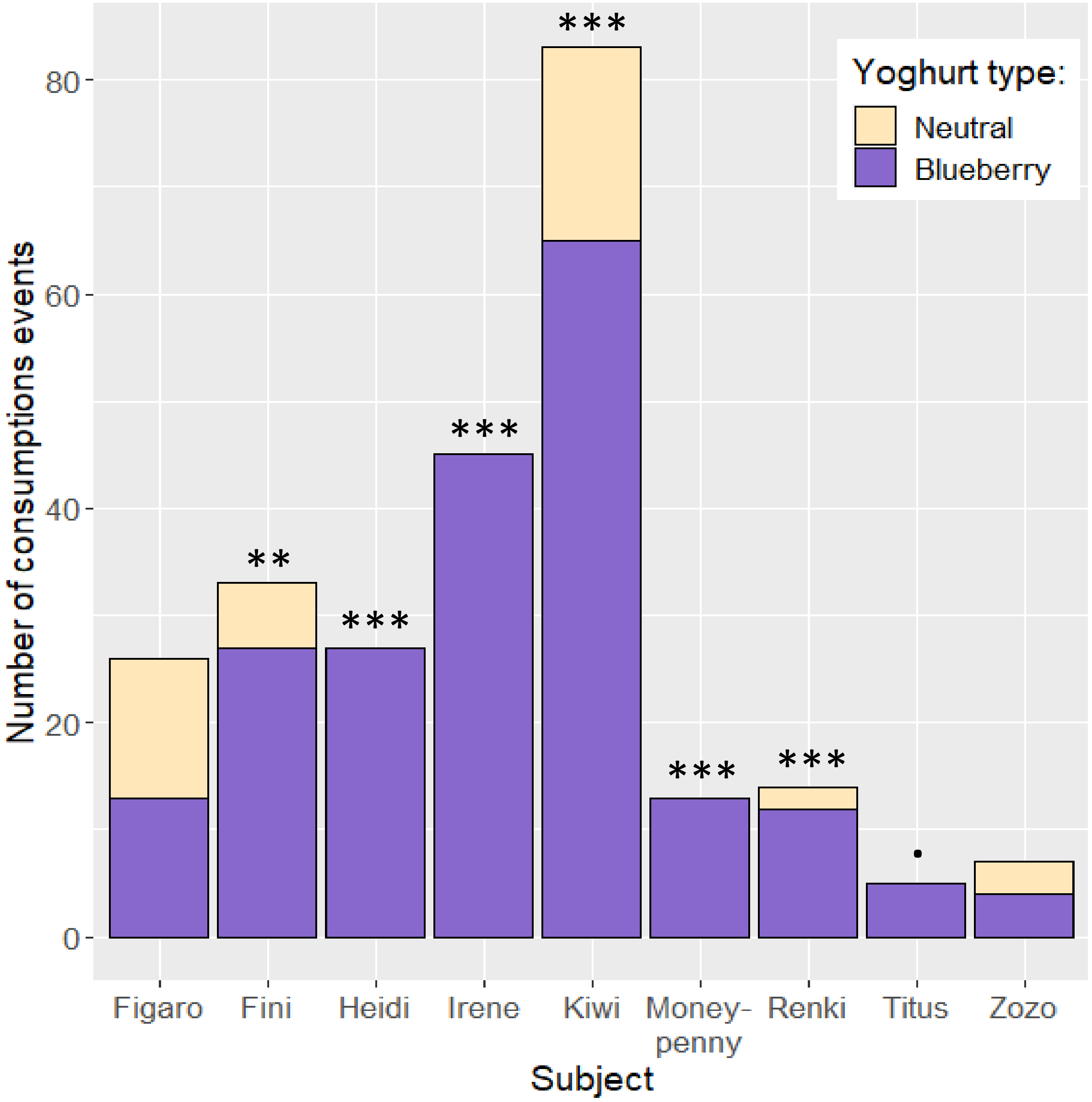


**Figure S2. Yoghurt preferences for each individual. Related to Figure 1.**
The total number of consumption events of the yoghurt only eating during all breakfast sessions. The colour represents the yoghurt eaten. The significances of the individual preferences are represented with: . p<0.1, * p<0.05, ** p<0.01, *** p<0.001.

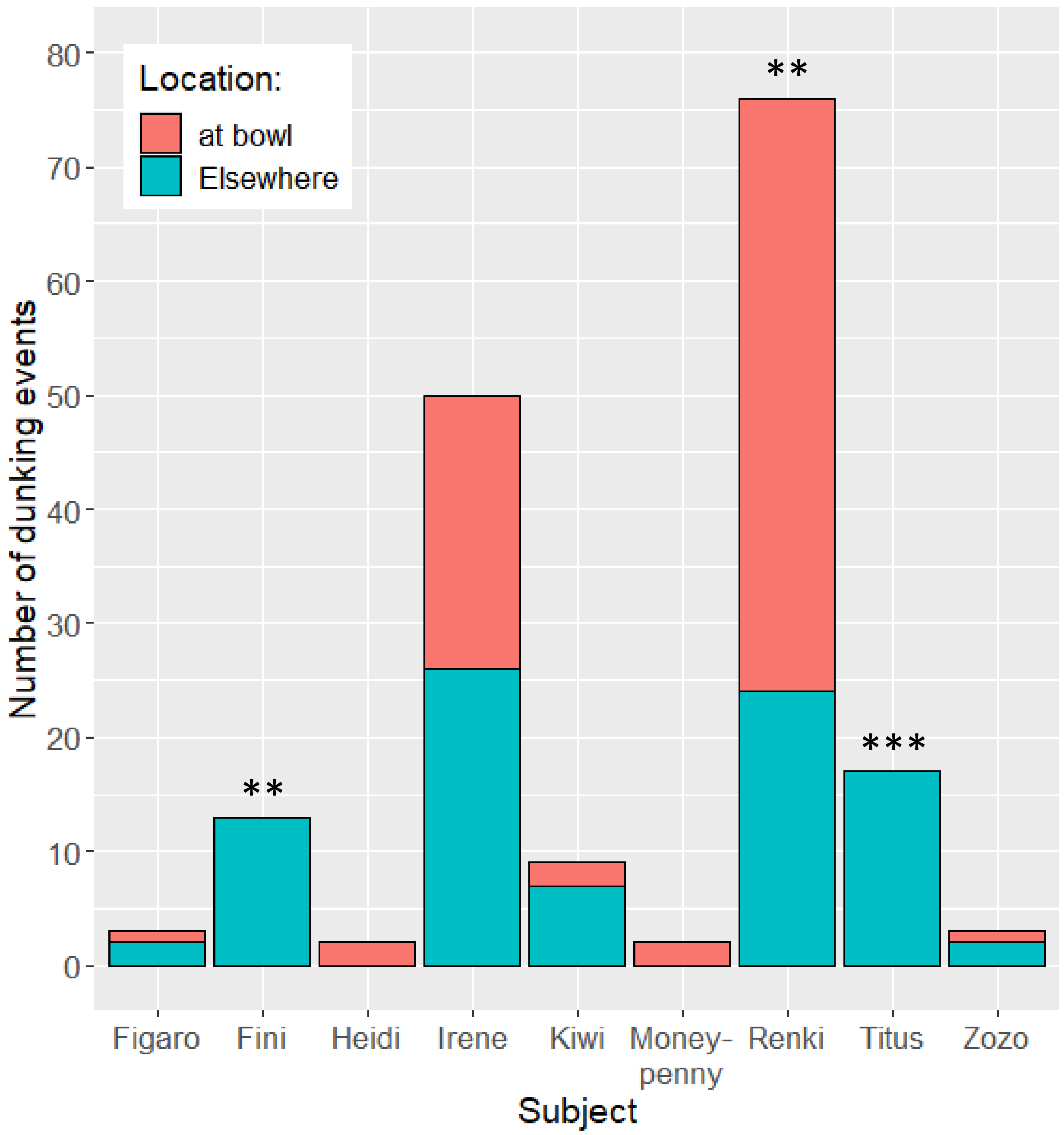


**Figure S3. Location where food was eaten after dunking for each individual. Related to Figure 1.** The total number of consumption events of the dunked food during all breakfast sessions. The colour represents where the food was eaten. The significances of the individual preferences are represented with * p<0.05, ** p<0.01, *** p<0.001.

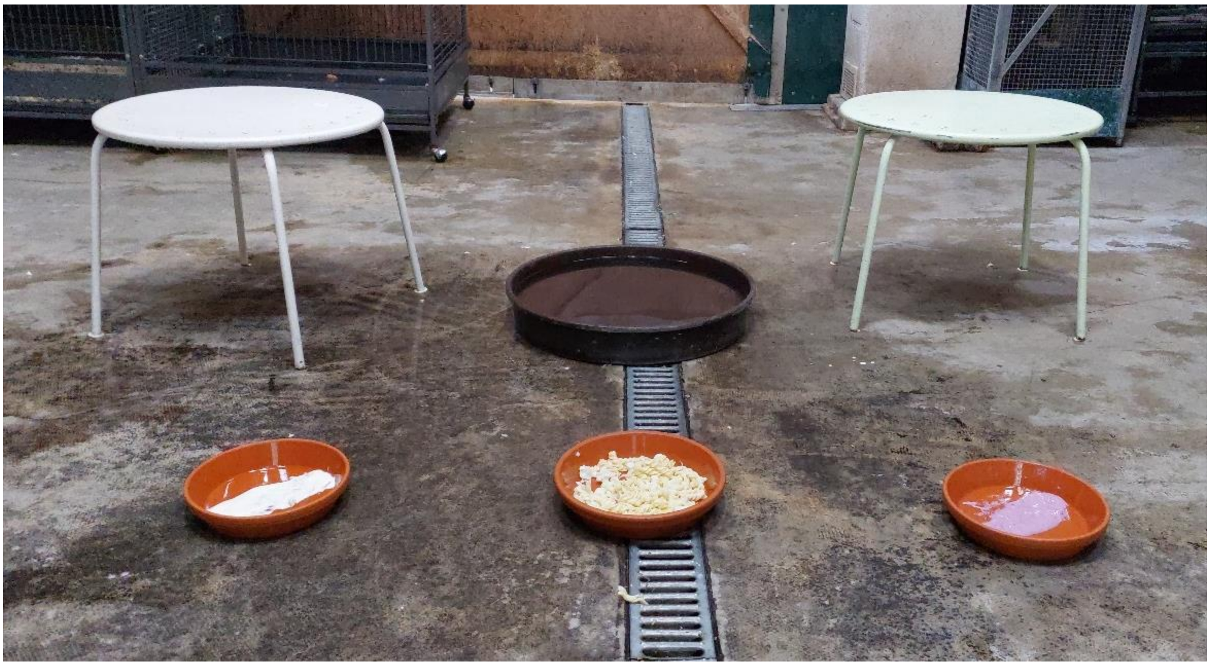

**Figure S4. Picture of the observation setup. Related to STAR methods and Figure 1.**
The food bowl (either with cauliflower and noodles or carrots and potatoes) places in the middle and at equal distances the water bowl (back), the neutral yoghurt (left) and the blueberry yoghurt (right). The position of the yoghurts were randomised over sessions.

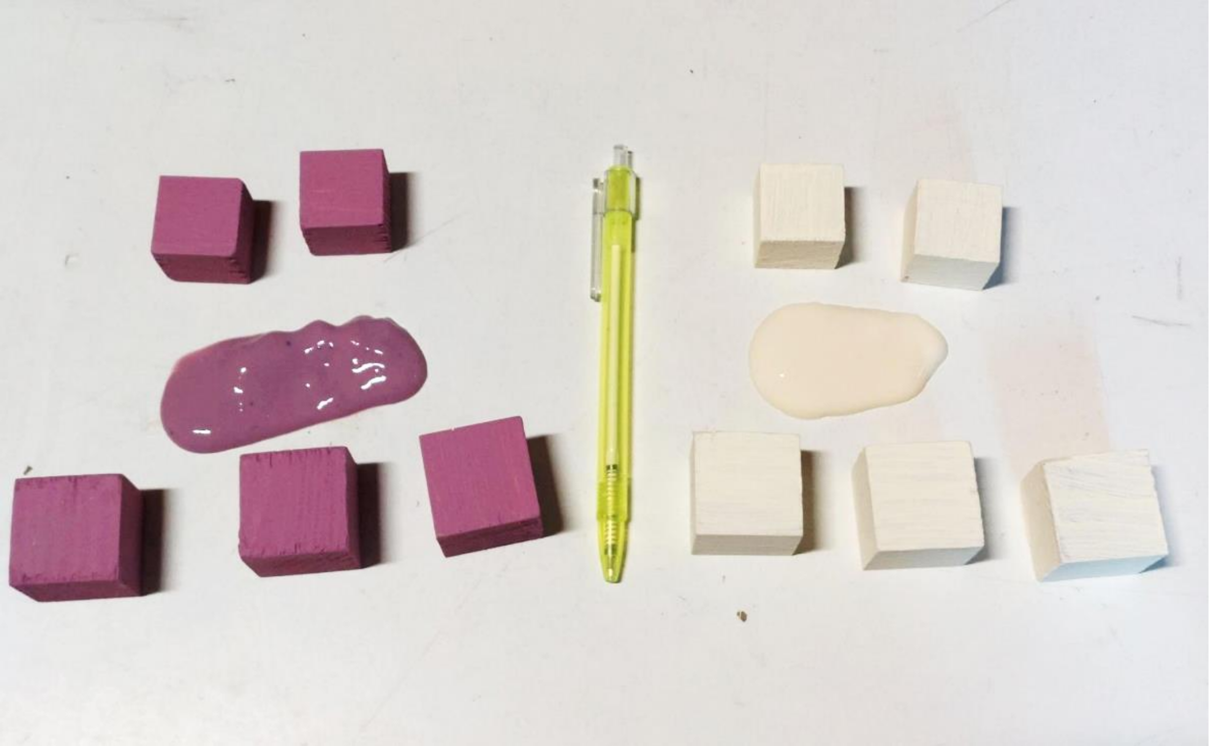

**Figure S5. The coloured cubes in comparison with the colour of the blueberry yoghurt (left) and the neutral yoghurt (right). Related to STAR methods**.

| Individual | Sex | Age (y) | Total number of dunking events | Dunked in Zewald & Auersperg (2023) | Time food left in yoghurt (s) | |
|---|---|---|---|---|---|---|
| | | | | | Average | sd |
| **Doolittle** | ♂ | 12 | 0 | No | - | - |
| **Doree** | ♀ | 2 | 0 | Yes | - | - |
| **Figaro** | ♂ | 16 | 3 | No | 1,67 | 1,06 |
| **Fini** | ♀ | 16 | 12 | No | 4,18 | 3,25 |
| **Heidi** | ♀ | 13 | 2 | No | 1,56 | 0,09 |
| **Irene** | ♀ | 6 | 49 | No | 3,32 | 2,85 |
| **Jane** | ♀ | 6 | 0 | Yes | - | - |
| **Kiwi** | ♂ | 13 | 9 | Yes | 3,05 | 1,77 |
| **Mayday** | ♀ | 12 | 0 | No | - | - |
| **Moneypenny** | ♀ | 13 | 2 | Yes | 3,40 | 1,93 |
| **Muki** | ♂ | 12 | 0 | Yes | - | - |
| **Muppet** | ♂ | 13 | 0 | No | - | - |
| **Olympia** | ♀ | 13 | 0 | No | - | - |
| **Pippin** | ♂ | 15 | 0 | Yes | - | - |
| **Renki** | ♂ | 3 | 72 | No | 3,69 | 3,97 |
| **Rosy** | ♀ | 2 | 0 | Yes | - | - |
| **Titus** | ♂ | 6 | 17 | No | 4,38 | 4,21 |
| **Zozo** | ♂ | 13 | 3 | No | 3,59 | 1,44 |

**Table S1. Subject details. Related to Figure 1 and STAR methods.**

The subject's name, age, number of food dunks, whether they dunked in the previous study and the average time ± standard deviation a subjects left the food in the yoghurt before eating.

| Behaviour | Point/State | Description | Extra modifiers |
|---|---|---|---|
| Take food | Point | The bird takes food from the food bowl or floor with its beak | **Food item**: Potato, Carrot, Cauliflower, Noodle<br>**Food eaten?**: Yes, dry; Yes, dunking in yoghurt; No |
| Dunk food | State | The bird puts a food item in a medium before consuming it (adapted from Morand-Ferron *et al.*, 2006[1]) | **Food item**: Potato, Carrot, Cauliflower, Noodle<br>**Dunking medium**: Water, neutral yoghurt, blueberry yoghurt<br>**Occupation at other bowl**: None, Bird name, Multiple<br>**Food eaten?**: Yes, No<br>**Eating where**: At bowl (<20cm), elsewhere (>20cm) |
| Dunk food again | State | The bird puts a food item in a medium after it already (partially) ate the food. | **Food item**: Potato, Carrot, Cauliflower, Noodle<br>**Dunking medium**: Water, neutral yoghurt, blueberry yoghurt<br>**Occupation at other bowl**: None, Bird name, Multiple<br>**Food eaten?**: Yes, No |
| Eat yoghurt | Point | The bird consumes yoghurt | **Yoghurt type**: Blueberry yoghurt, Neutral yoghurt<br>**Occupation at other bowl**: None, Bird name, Multiple |
| Bowl proximity | State | The subject comes within one body length near the one of the bowls | - |

**Table S2. Ethogram. Related to STAR methods.**

The behaviour, whether it was recorded as a point (event) behaviour or a state (duration) behaviour, its description and the extra modifiers recorded every time this behaviour occurred.
